# Profile. The constant psychiatrist: an interview with Michael Kopelman

**DOI:** 10.1192/pb.bp.116.055590

**Published:** 2017-12

**Authors:** Norman Poole

From his journal-lined office in St Thomas' Hospital Professor Michael Kopelman could quietly yet assiduously observe the powerful in their Palace of Westminster just across the Thames. Following retirement in 2015 he has become ever more alarmed by the spectacle:

‘Our society, in my view, is becoming more and more authoritarian. It's very worrying the direction we are moving in. Even within our university system and the NHS things have become much less democratic than they used to be, and much more authoritarian. It's both sad and frightening.’

A perpetually crumpled mac under his arm, the Professor appears much like an MI6 officer in a le Carré novel grown disillusioned with the culture of edicts, diktats and target-driven performance for the workers while those in positions of power sit removed and aloof.

‘Now it's really the chief executive and his executive team who run things. With no disrespect to any particular individual, some of whom are very good, I can't see that they have proper accountability. We doctors have accountability. We can be hauled before the GMC. But managers and commissioners, who are changing the healthcare environment, often for the worse, are not held to account in the same way.’

The Professor's comportment is at one with his assertion, ‘These are matters I feel strongly about.’

Indeed. For just as the anti-heroes in le Carré's novels battle with the bureaucracies in which they work, Professor Kopelman is exasperated by perceived wisdom, particularly with regard to a matter close to his professional heart, the modern memory clinic. ‘The memory clinics that are being set up under the Dementia Strategy are not what I would advocate.’ A Fellow of the Academy of Medical Sciences and founding member of the Memory Disorders Research Society, Professor Kopelman appreciates the diversity of problems that have an impact on cognition.

‘There is a wide range of memory disorders to be diagnosed, and the early diagnosis of dementia, even by the very experienced, is difficult. There are very sad implications if you get it wrong. The earlier we go for diagnosis the more likely we are to get it wrong, whatever the clinical/genetic/biomarker tools that we have.’

Criticising the consensus again, he says:

‘I think, and I'm not popular for saying this, that nurse-led diagnostic teams making early diagnosis is not the direction to go in. The direction to go is better care for those with established diagnoses of dementia. What we have at present is shameful, and in my view it's actually now worse than in the 1980s.’

**Figure:**
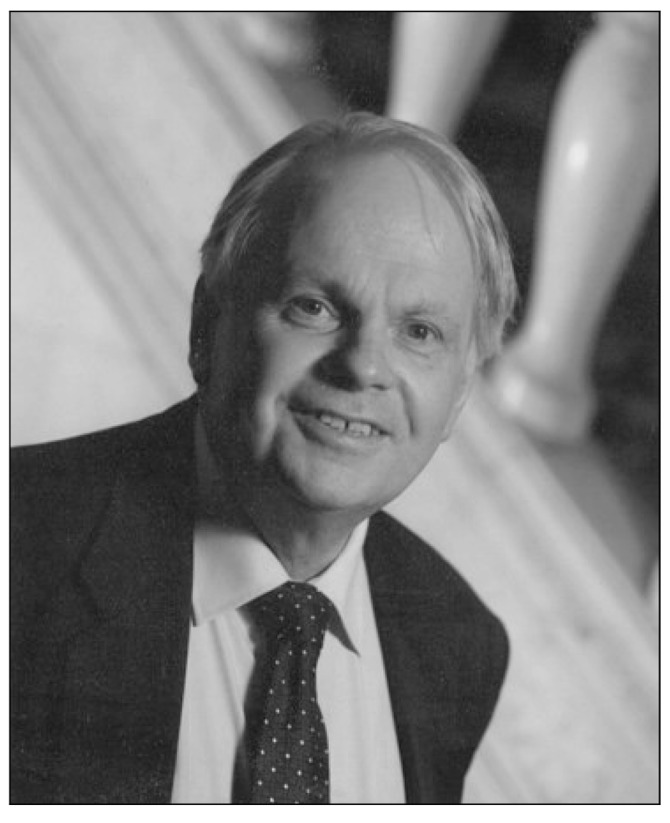
Professor Michael Kopelman.

It is perhaps this disillusionment that partly drives his medico-legal work, in which he is fearless in tackling injustice head-on. On many occasions he has been involved in headline-grabbing courtroom dramas that could themselves be the stuff of fiction. Interestingly, he highlights a radical human rights lawyer as the anti-establishment role model for his own legal work.

‘Gareth Peirce is superb at using the legal rules to beat authoritarians and government. She plays within the system, but she does it better than the government lawyers and beats them. That fits my temperament. Not shouting or protesting on the streets, but playing the system to get justice for people.’

Clashes with the establishment have seen Professor Kopelman fight for the falsely convicted and for Guantánamo detainees. He said:

‘I got into false confession cases, which I see as a form of memory disorder, and was involved in overturning convictions; one from 50 years ago, another after 25 years, and a delusional memory case at 26 years.’

He added:

‘Then with two others – a therapist and a GP – I wrote a report in 2010 on people who had come back from Guantánamo, including some prominent names, and this resulted in them getting substantial amounts of compensation. Ken Clarke announcing this in Parliament made the somewhat ambiguous statement, “We must never let this happen again.” I feel in some ways I have done more good from this sort of work than anything else, and that's what I'm going to do in my retirement.’

His colleagues obviously agree as Professor Kopelman has previously been elected to serve as president of the British Academy of Forensic Sciences.

This ability to perform scalpel-sharp analyses of intricate legal cases and their relation to esoteric psycho-pathology originates in his wide-ranging reading list at medical school and a first degree in psychology. ‘I had gone into psychology and enjoyed it, and before that – oh I hate to say the cliché – I was interested in people, and enjoyed literature from the psychological angle. So this is what I was curious about.’ Upon gaining a place studying medicine at Middlesex University, he was not your typical medical student: ‘I read Luria quite early on, when I should have been reading anatomy textbooks!’

Paradoxically, a contemporary model of memory claims that it evolved to enable planning for the future, or mental time travel.^1^ Professor Kopelman's forays into the science of memory were thus doubly prescient:

‘I'd been interested in the neuropsychology of memory, which was just developing at the end of the 60s and 70s. I was reading quite a lot of the amnesia stuff at an early stage and I was interested in the more biological aspects of psychology. I knew I would either do neurology with an interest in cognitive neurology or psychiatry with an interest in neuropsychiatry, and I've ended up in the middle discipline.’

That's rather too modestly put given that Professor Kopelman actually ended up as president of both the British Neuropsychological Society and the International Neuropsychiatric Association, and is currently Presiding President of the International Neuropsychological Society.

The influence of psychology so early in his academic career may well contribute to his continued aversion to reductive thinking about mental disorder: ‘I get a bit anxious about what I call naive reductionism, the kind of approach that thinks that all PTSD and depression are just disorders of the hippocampi or the frontal lobes or whatever. I think that is very simplistic.’ Adding, ‘We have to remember the brain is operating within a social context and not to underplay the importance of our social context.’ It is to his own social context that his mind now turns. Between confrontations with government barristers and completing research programmes at St Thomas', where he can still be found a few days of the week, Professor Kopelman divides his time between a beautiful 18th-century home in Surrey and a cottage in Norfolk. It is apparent that similarities to le Carré's heroes don't just end with the raincoat.

## References

[1] Michaelian K Mental Time Travel: Episodic Memory and Our Knowledge of the Personal Past. MIT Press, 2016.

